# Efficacy of a whole-body vibration intervention to effect exercise tolerance and functional performance of the lower limbs of people with chronic obstructive pulmonary disease

**DOI:** 10.1186/1471-2466-12-71

**Published:** 2012-11-26

**Authors:** Trentham Furness, Nicole Bate, Liam Welsh, Geraldine Naughton, Christian Lorenzen

**Affiliations:** 1 Department of Medicine, Monash University, Monash Medical Centre, Clayton, Australia; 2 School of Exercise Science, Australian Catholic University, Melbourne, Australia; 3 Monash Medical Centre, Southern Health, Clayton, Australia; 4 The Royal Children’s Hospital, Melbourne, Australia

**Keywords:** Whole-body vibration, Chronic obstructive pulmonary disease, Functional performance

## Abstract

**Background:**

Chronic obstructive pulmonary disease (COPD) is a respiratory condition characterised by dyspnoea, excessive sputum production, chronic cough, bronchitis and emphysema. Functionally, exercise tolerance is poor for people with COPD and is linked to difficulty in performing daily tasks. More specifically, exercise difficulties are due partly to dyspnoea and lower limb skeletal muscle dysfunction. The benefit of exercise that does not exacerbate the disease while improving exercise tolerance is salient. Whole-body vibration (WBV) is a mode of physical activity known to improve muscular function of the lower limbs, yet efficacy has not been investigated for a WBV intervention conducted in a home-based setting for people with COPD.

**Methods/design:**

This clinically registered trial is a non-randomised placebo cross-over intervention based in the home of each participant (ACTRN12612000508875). Participants diagnosed with COPD will complete a six-week WBV intervention and then after a two-week washout period, will complete a six-week placebo training intervention. Participants will complete sessions twice a week. The duration of the trial is 14 weeks. Community-dwelling older adults with COPD will provide informed voluntary consent to participate. Outcome measures will include immediate, acute, and long-term responses to exercise.

**Discussion:**

Quantifying responses to WBV among people with COPD will allow discussion of efficacy of WBV as a mode of physical activity. The skill required by the participant to perform physical activity with WBV is not demanding and may enhance habitual sustainability. The results of this trial could be used to support further research in both clinical and community settings.

**Trial registration:**

Australian New Zealand Clinical Trials Registry (ANZCTR12612000508875)

## Background

The World Health Organisation’s (WHO) current definition of chronic obstructive pulmonary disease (COPD) is a lung ailment fundamentally characterised by persistent blockage of airflow from the lungs [1]. In 2004 the WHO estimated 64 million global cases of COPD and later predicted COPD to become the third leading cause of death by 2030 [2]. Among Australians, COPD was the third leading cause of burden of disease and injury behind ischaemic heart disease and stroke [3]. Somewhat understandably, tobacco usage was listed with physical inactivity as the two leading risk factors eliciting burden of disease in Australia [3]. Although not always the case, tobacco smoke is the leading risk factor for COPD [4]. While the cost of COPD in Australia has not been directly estimated, respiratory disease alone accounted for AU$3.31 billon (6.3%) of the total allocated health expenditure in Australia [5].

Given that COPD diminishes the ability of the lungs to supply the body with oxygen, most modes of physical activity lead to breathlessness if the disease is progressed. Perceived breathlessness, or dyspnoea, leads to physical inactivity and consequently compounds the risk of burden of disease to those individuals. As a counteractive strategy, physical activity is routinely incorporated to the management of stable COPD [6].

Recommendations from the WHO, the American Thoracic Society, the European Respiratory Society and the Thoracic Society of Australia and New Zealand, and other professional bodies support physical activity as integral to pulmonary rehabilitation programs in public heath settings to assist individuals affected by COPD [6]. A goal of physical activity within pulmonary rehabilitation is to improve exercise tolerance for people with COPD. During physical activity, the cardiovascular and neuromuscular systems are stressed above resting levels, which if continued over time, can improve systemic responses. Specifically, an improvement of cardiac output and cellular respiration are common after physical activity interventions [7]. Improvement in exercise tolerance has functional benefit such as the increased ability to complete activities of daily living (ADLs).

Maintenance and improvement of ADLs is central for functional independence [6,8]. Given that functional independence can rely on the ability to complete ADLs, muscular strength, muscular power and gait ability are considered important. Activities requiring strong and powerful muscular contraction are fundamental and include rising from a chair, climbing stairs, turning and adjusting posture, and stumbling to avoid a fall. People with COPD have less muscular strength and power than healthy age matched controls [9]. Subsequently, this population may have poorer exercise tolerance associated with muscular weakness [10]. As such, for people with COPD, muscular deconditioning/dysfunction is postulated to lead to poor performance of ADLs and poor exercise tolerance.

A primary objective of pulmonary rehabilitation is to combat poor muscular performance. The components of pulmonary rehabilitation vary, but include physical activity (both aerobic conditioning and resistance training), healthy lifestyle education, and nutrition counselling [11]. Initially, pulmonary rehabilitation can be implemented at an outpatient setting, usually followed by a home-based intervention that relies mostly on the compliance of the patient. Efficacy was established after systematic review of both outpatient pulmonary rehabilitation [12-14] and home-based pulmonary rehabilitation [15-17].

During pulmonary rehabilitation, aerobic conditioning was most effective in improving exercise tolerance at intensities over 50% of peak oxygen consumption (VO_2peak_) [11,18]. Although resistance training improved muscular strength [19], muscular power [20] and variables of gait [21], dedicated strategies for resistance training of the lower limbs lack thorough condition specific recommendation for people with COPD. A recent literature review of resistance training during peripheral muscle training reported the efficacy of resistance training for people with COPD [8]. As such, resistance training of the lower limbs elicited appreciable gains in muscular strength for people with COPD that may carry over to performance of some ADLs [8]. However, resistance training was conducted in conjunction with other modes of physical activity such as aerobic conditioning. The independent effect of resistance training therefore, could not be established.

Considering the statistical and scientific evidence for efficacy of physical activity to reduce burden of disease, the need for safe and valid exercise interventions for people with COPD is salient. The common modes of physical activity; aerobic conditioning and resistance training can exacerbate the disease and affect program compliance [22,23] and may lead to reduced physical activity because of fear of breathlessness. The clinical and social merit of physical activity that can minimise dyspnoea may provide additional exercise tolerance benefit for people with COPD. Whole-body vibration may be such a mode of physical activity, and can be easily completed in the home with little skill demand.

Whole-body vibration is a mode of physical activity during which an individual stands on a vibration platform that can create acceleration predominantly in the vertical (Fz) direction. The accelerations are transmitted to the body and postulated to elicit physiological responses similar to other modes of physical activity such as aerobic conditioning and resistance training.

After WBV, increased leg muscular strength and muscular power [24,25], oxygen consumption [26,27], growth hormone, and testosterone levels [24,28] were reported in healthy young and older adult populations. Furthermore, WBV may be a safe and effective mode of physical activity to improve muscular strength, body balance and mechanical competence of bone for older adults with low bone mineral density [29,30]. A growing body of literature on the impact of WBV has included sub-optimal health populations such as cystic fibrosis [31], multiple sclerosis [32] and stroke [33]. Yet to date, well conducted trials into the efficacy of WBV in patients with COPD are scarce in the literature.

One paper has been published to investigate efficacy of WBV to improve muscular strength and muscular power of people with COPD. In conjunction with a clinically based three-week pulmonary rehabilitation intervention, WBV may have enhanced exercise tolerance [34]. The authors suggested that long-term studies are needed to determine optimal intensity and duration of WBV for people with COPD, though WBV seemed to be a promising new mode of physical activity [34]. However, efficacy of a standalone WBV intervention remains unknown in outpatient and home-based settings.

### Objective

The objective of the trial is to advance knowledge of effects of physical activity on exercise tolerance and functional performance of the lower limbs of people with COPD, and to test the efficacy of a WBV intervention to beneficially effect exercise tolerance and functional performance of the lower limbs without causing exacerbations. The intervention will be conducted and data collected in the home of each participant.

## Methods/design

This trial will be conducted as a non-randomised intervention with crossover to placebo. Given the nature of COPD, and exacerbations expected of it, this trial will be a phase II efficacy trial and as such will not be conducted as a randomised controlled trial. People with stable COPD will be invited to participate. It is necessary to first conduct the WBV intervention, and then the PLACEBO intervention in an effort to maximise participant numbers in the WBV group, with anticipated dropout over the course of the 14-week intervention. A control condition could compromise trial compliance and randomisation is not critical to an efficacy trial.

### Participants and recruitment

Community-dwelling patients listed on the Department of Respiratory and Sleep Medicine, Monash Medical Centre, Southern Health data-base will be contacted to procure interest in participation and later provide informed voluntary consent. Participants must be over 40 years of age, be fully independent in residence, and have well-managed, stable Stage II COPD according to the Global initiative for chronic Obstructive Lung Disease (GOLD) criteria. A convenient sample of people with stable Stage II COPD will be targeted due to a reduced chance of participant dropout from exacerbations independent of the 14-week intervention. Participants must be free of contraindicators to physical activity as well as contraindicators to WBV (free of self reported; vascular disease, reactive arthritis, vertigo, and risk of thromboembolism [28,35,36]. The trial has the ethical approval of the Monash Medical Centre, Southern Health Human Research Ethics Committee A and the Australian Catholic University Human Research Ethics Committee. The trial is registered with the Australian New Zealand Clinical Trials Registry (ACTRN12612000508875).

### Interventions

Across the 14-week duration of the trial, participants will be first allocated to a six-week WBV intervention, a two-week washout period [37], and a six-week PLACEBO intervention.

### WBV intervention

The WBV intervention will consist 12 WBV sessions over a consecutive six-week period. The participants will complete two vibration sessions per week separated by at least 48 hours. Efficacy of session frequency had been previously established for healthy community-dwelling older adults [25]. A WBV session will consist of five, 60 second vibration bouts, interspersed with 60 seconds of passive rest. Although the most effective protocol is yet to be established, the selected protocol had been used previously to establish efficacy of WBV interventions [36,38,39]. During rest, the participant will remain on the vibration platform and will be encouraged to stand with a posture that resembles the ‘anatomical position’. More specifically, the participant assumes a standing position with the feet together, the arms to the side, and the head, eyes and palms facing forwards. The forearms are supinated.

A side alternating vibration platform (Amazing Super Health, Melbourne, AUS) will be used. For each vibration bout, platform frequency will be 25 Hz, peak-to-peak displacement 2.0 mm, peak acceleration ~24.67 m.s^-2^, and gravitational force ~2.52 *g*. The WBV intensity was chosen to reduce the risk of fracture to fragile bones [40] and potential for other injury [41]. The vibration platform peak-to-peak displacement and frequency were validated prior to this proposal in an unpublished pilot study. Foot placement (second toe) will be equidistant, 20 cm from axis of rotation. The participant will wear flat soled shoes. Skidding will be checked according to the recommended method of the International Society of Musculoskeletal and Neuronal Interactions. A piece of paper will be placed under the foot during a vibration bout to ensure constant contact with the vibration platform [42]. The participant will be required to stand with ~20º knee flexion to allow transmission of vibration about the lower limbs [43]. Knee angle will be filmed, and later quantified across the intervention for each participant.

### PLACEBO intervention

A prototype vibration platform will be used for the PLACEBO intervention. For each vibration bout, platform frequency will be 25 Hz, peak-to-peak displacement ~0.0 mm, peak acceleration ~0.00 m.s^-2^, and gravitational force ~0.0 *g* (cognisant that the Earth’s gravitational force is constant 1.0 *g*). Participants will be required to follow the same procedure of the WBV intervention. Participants will be told the PLACEBO intervention is an “ultra-low frequency” vibration intervention that is “very different” to the aforementioned WBV intervention.

### Outcome measures

Reliability of outcome measures will be established over a test-retest-retest protocol. Exercise tolerance variables will be immediate responses to WBV; (1) perceived dyspnoea quantified with the Borg CR-10 visual analogue scale, (2) heart rate, and (3) saturation of haemoglobin. Functional performance variables will be simulated ADLs and kinematic markers of gait; (1) the 5-chair stands test, (2) the timed up and go test, (3) stride length, (4) stride time, and (5) stride velocity. Data will be collected at baseline and subsequent fortnight for 14 weeks. Data of all dependent variables will be collected to quantify at least one of three different data classifications: (1) immediate, (2) acute, and (3) long-term. Immediate perceived dyspnoea, heart rate, and saturation of haemoglobin will be collected during the final WBV or PLACEBO bout (after 30 seconds of the 60 second bout). Acute kinematic markers of gait will be collected within two minutes of a completed WBV or PLACEBO bout [38,44]. Long-term simulated ADLs and kinematic markers of gait will be collected at least 48 hours after a WBV or PLACEBO bout [25,45].

To complete the 5-chair stands test the participant is asked to sit in a standard height (46 cm, including back rest) chair with arms folded across the chest. The participant is then asked to stand and sit five times [35,46].

To complete the timed up and go test, the participant is asked to sit in a standard height (46 cm) arm (63 cm) chair. The participant is then asked to stand, walk three metres to a marker on the floor, turn, return, and sit on the chair [46,47].

Kinematic markers of gait will be quantified with the GAITRite® electronic walkway (CIR Systems Inc, Peekskill, USA). Participants will be instructed to walk at a self-selected comfortable speed along a straight, flat, and even walkway area containing the mat. Body length is measured from the base of the foot (unshod) to the crown of the head while standing in the anatomical position. During data collection, participants will walk (shod) over the mat five times in a discontinuous process with an approach and departure distance equal to two body lengths. The procedure was reliable for step length, step time, and velocity among older woman (ICC range 0.860 to 0.940; mean age = 68 years) [48].

### Sample size

It is expected that WBV will not change dyspnoea from baseline. To allow sample size calculation therefore, data of functional performance variables were considered primary and used to estimate sample size. The selected dependent variables were sourced from a previous study of WBV and healthy community-dwelling older adults [25]. Sample size needed for a one tailed paired *t*-test with α 0.05 and *n* = 14 with power = 0.90 was *n* =16 for the 5 chair stands test *n* < 10 for the timed up and go test. To allow for an anticipated participant drop out of at least 20%, a total of 20 participants will be included in the study.

## Discussion

Whole-body vibration is a mode of physical activity thought to affect the musculoskeletal, endocrine and cardiovascular systems of the human body. Researchers can choose to use several platform types, a range of vibration frequencies, peak-to-peak displacements and a multitude of stance postures to affect dependent variables. As such, both effective and ineffective results from WBV have been reported. However, negative effects of WBV are rarely reported when vibration platforms and protocols are matched to a specific population [49]. Considering the growing WBV literature base, it is an oversight that prescription guidelines for safe and effective WBV have not been established, despite increasing existence of vibration platforms in the health and fitness industry. However, since many variables of a WBV intervention can be manipulated, comparison among studies and subsequent guideline recommendations are difficult.

Although WBV has rarely been used as an exercise intervention for people with sub-optimal health, condition specific exercise is possible. Whole-body vibration had limited use as an exercise intervention for people with COPD. However, from the paucity of available literature base on sub-optimal health samples, WBV exacerbations are rarely described, but may lack full disclosure.

A WBV intervention should be safe and valid before implemented to a sample cohort, regardless of health status. Encouragingly, WBV did not negatively affect people with stable COPD [34], people with cystic fibrosis [31] or heart transplant recipients [50]. Despite current knowledge of WBV, as a standalone home-based mode of physical activity, efficacy has not been established for improvement or maintenance of exercise tolerance and functional performance of the lower limbs of people with COPD. Furthermore, it is unknown if a long-term WBV intervention will elicit exacerbations of COPD, or be well tolerated and complied by people with COPD.

## Competing interests

The authors declare that they have no competing interests.

## Authors' contributions

All authors have made substantial contributions to conception and design and potential acquisition of data. All authors have been involved in drafting the manuscript or revising it critically for important intellectual content. All authors have given final approval of the version to be published.

## Pre-publication history

The pre-publication history for this paper can be accessed here:

http://www.biomedcentral.com/1471-2466/12/71/prepub
